# MAternal Dietary changEs (MADE) Study Protocol: A Mixed‐Methods Study of Breastfeeding Mothers in England

**DOI:** 10.1111/cea.70356

**Published:** 2026-05-26

**Authors:** Anna Gilbertson, Matthew J. Ridd, Robert J. Boyle, Raquel Granell, Joanna Kesten

**Affiliations:** ^1^ Centre for Applied Excellence in Skin & Allergy Research, Bristol Medical School University of Bristol Bristol UK; ^2^ School of Public Health, Faculty of Medicine, Imperial College London UK; ^3^ The National Institute for Health and Care Research Applied Research Collaboration West (NIHR ARC West) University Hospitals Bristol and Weston NHS Foundation Trust Bristol UK; ^4^ NIHR Health Protection Research Unit in Evaluation and Behavioural Science, Bristol Medical School University of Bristol Bristol UK

## Abstract

- Maternal dietary changes influenced by allergy during breastfeeding are not well evidenced or informed.
- This mixed‐methods study aims to estimate the prevalence and impact of maternal dietary changes during breastfeeding.

To the Editor,

A well‐balanced diet is important for breastfeeding mothers and a varied diet may be enjoyed without restrictions. However, widely held beliefs that foods in a mother's diet may cause symptoms suggestive of illness in the breastfed infant are not new and dietary changes, such as restricting foods perceived to cause gastrointestinal discomfort, are common across cultures [1]. Associations between infant symptoms and food allergy are not new either, but increased awareness or concerns about food allergy are influencing the changes mothers make during breastfeeding [2].

Cow's milk allergy is commonly said to present a diagnostic challenge in community practice, where infants often present with non‐specific symptoms such as excessive crying, skin rashes, vomiting or stool changes [3]. These early infant symptoms are common regardless of how they are fed or whether they develop cow's milk allergy (which may be IgE or non‐IgE mediated or both). Formula industry influence on symptom‐based guidance has contributed to controversy over current guidance, and practice, for detecting and managing cow's milk allergy [3]. Consequently, cow's milk allergy may be attributed to common gastrointestinal symptoms for many infants without cow's milk allergy and, based on increased usage of specialised formula, there are concerns that it is over‐diagnosed [4, 5].

Since cow's milk allergy is an immune system response to the ingestion of specific cow's milk proteins, ingestion of milk proteins by breastfed infants must be indirect, via maternal consumption of cow's milk. Current guidance for managing symptoms attributed to cow's milk allergy often involves strict maternal dietary restriction of dairy (and potentially other foods) [6]. Advice to reintroduce the food after a period of exclusion is necessary to confirm sensitisation. Whilst continuation of breastfeeding is usually, and should always be encouraged, some guidelines recommend providing specialised formula to supplement or replace breastmilk [6]. However, investigations of food protein transfer through breastmilk suggest that, for many breastfed infants with symptoms, maternal dietary restrictions (or specialised formula) may be unnecessary, may be harmful, and should not be promoted [2, 3, 7].

Nevertheless, only a small range of foods and food allergens have been studied in this context and, for breastfeeding mothers with concerns about an infant's symptoms, changing their own diet may seem a harmless, safe and easy intervention. This is particularly true when they believe there is a direct relationship between their infant's symptoms and what they are eating. There is a danger, when conditions are difficult to diagnose, have contested opinion and/or ambiguous symptoms, that patients can feel dismissed and unsupported by healthcare professionals [8]. The role of the healthcare professional is to acknowledge concerns and offer support with their decision‐making [2] but often changes are made without careful planning or regard for the mother, their well‐being or the importance of maintaining a well‐balanced diet during breastfeeding.

It is not known how commonly mothers restrict their diet due to allergy concerns and what impact this has on their own health, their confidence with other foods in their diet or with their breastfeeding journey. Whilst concerns have been raised about potential harms of promoting maternal dietary restrictions for allergy management, there are also potential benefits, and this has not been a research priority [2].

Hence, we are conducting the MAternal Dietary changEs (MADE) study (Figure 1), We will use mixed methods to explore the prevalence and nature of maternal dietary changes during breastfeeding and the impact, from mothers' perspectives, when the changes are specifically restrictions for managing infant symptoms [9]. In short, mothers in England will be invited by their primary care practitioner to take part in an online survey, which will ask what, if any, changes they have made to their diet during breastfeeding, what the reasons are for the changes, and any factors influencing decision‐making. Mothers will be eligible to participate if their child is under 12 months old and they have breastfed them, by any means, for any duration. We will aim for geographical and deprivation level diversity by engaging with at least 24 primary care practices via Regional Research Delivery Networks across England. We estimate that a sample size of 800 mothers will have 80% power to detect a minimum prevalence difference of 10% between groups such as age and parity.

**FIGURE 1:**
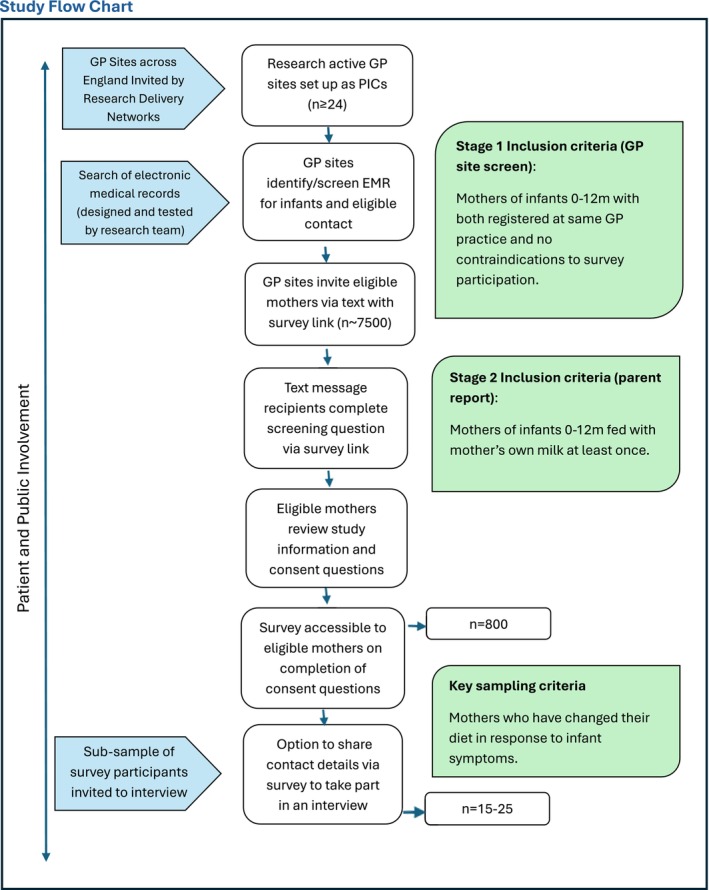
Study flow chart.

Next, a group of mothers who complete the survey and report changing their diet in response to infant symptoms will be invited to be interviewed. We will conduct approximately 25 semi‐structured, one‐to‐one interviews using a topic guide to explore whether symptoms attributed to allergy influenced the dietary changes and the nature of any advice or support regarding the removal and reintroduction of foods from their diet. The perceived impact of dietary changes on their wellbeing and breastfeeding confidence will also be explored. Analysis will use a combination of deductive and inductive coding approaches to generate themes from the mothers' responses to the topics raised by the interviewer. The total number of interviews will be determined iteratively and pragmatically.

Maternal dietary changes in response to infant symptoms may be increasing and do not have a strong scientific evidence base. There is controversy over the relative value or harm of maternal dietary changes during breastfeeding. At the end of this study, we will have characterised the prevalence and self‐reported impact of maternal dietary changes during breastfeeding in England. The study findings will better inform breastfeeding mothers, and those caring for them, when considering maternal dietary changes as an approach to managing infant symptoms.

## Author Contributions

A.G. conceived the study design and wrote the manuscript, with critical revisions from M.J.R., R.J.B., R.G., and J.K. All authors reviewed, commented, and approved the final manuscript.

## Funding

This research is funded by the National Institute for Health and Care Research (NIHR) as part of an NIHR Research Professorship (NIHR303123). J.K. is partly funded by the National Institute for Health and Care Research Applied Research Collaboration West (NIHR ARC West) and the NIHR Health Protection Research Unit in Evaluation and Behavioural Science. The views expressed are those of the authors and not necessarily those of the NIHR, the NHS, or the Department of Health and Social Care.

## Conflicts of Interest

R.J.B. declares funding from EAACI and BSACI for conference presentations, from Wiley and BSACI for editorial work, from the UK Department of Health and Social Care for expert advisory committee work, from WHO and the Norwegian Directorate of Health for consultancy and from legal firms for expert witness work related to food anaphylaxis and infant formula health claims.

## Data Availability

Data sharing not applicable to this article as no datasets have been generated or analysed yet.
